# Cell state programs offer a potential axis for classifying neuroendocrine tumors

**DOI:** 10.1172/JCI213026

**Published:** 2026-11-02

**Authors:** Parambir S. Dulai

**Affiliations:** Division of Gastroenterology and Hepatology, Northwestern University, Chicago, Illinois, USA.

## Abstract

Gastroenteropancreatic neuroendocrine tumors (GEP-NETs) arise from hormone-producing cells in the gut and pancreas. Despite increasing incidence of these tumors, therapeutic options remain limited. Well-differentiated GEP-NETs are classified by anatomic site, morphology, and proliferation indices, but this approach offers limited prognostic resolution among phenotypically similar tumors and constrains personalization of treatment decisions. In this issue of the *JCI*, Karam, Hoffman, and colleagues profiled 38 pancreatic and small-intestinal NETs using joint single-nucleus RNA and ATAC sequencing and projected the resulting malignant cell–associated programs onto matched spatial transcriptomes. Two conserved malignant states emerged: neuronal-like and secretory neuroendocrine. These states span a continuum in both organ sites, are enriched for largely different transcription factor motifs, and occupy distinct microenvironmental niches. Secretory program scores trended higher in metastatic disease and may carry prognostic value. Taken together, these data describe NET heterogeneity as a cell state axis linked to tissue architecture.

## The limits of tumor site and grade classification

Gastroenteropancreatic neuroendocrine tumors (GEP-NETs) arise from the hormone-producing cells dispersed through the gut and pancreas. Although they are relatively rare cancers, their incidence in the United States has risen several fold over recent decades (1, 2). For most solid tumors, rising incidence has been met with expanding therapeutic options coupled with personalization of treatment decisions through prognostic biomarkers and/or indices. For GEP-NETs, however, resection remains the only curative intervention, and somatostatin analogs, everolimus, peptide receptor radionuclide therapy (e.g., ^177^Lu-DOTATATE), and antiangiogenic tyrosine kinase inhibitors extend progression-free survival without producing cure (3, 4).

Well-differentiated GEP-NETs are assigned an organ of origin and a WHO grade (G1–3, with a higher number reflecting more aggressive tumors) based on a Ki67 proliferation index. However, it is common for two tumors identical by both criteria to diverge in progression, metastatic behavior, and treatment response. In a multisite analysis of 734 tissue sections, whether a tumor was called G2 depended on whether Ki-67 was counted in a hotspot or across the whole slide. Tumors with diffuse G2 staining showed significantly worse progression-free survival than those with focal G2 despite carrying the same WHO grade, and grades assigned at primary, regional, and distant sites did not consistently agree (5). Refinements to GEP-NET grading proposed to date have subdivided these same axes more finely, and genomics has not supplied a replacement (6, 7). Single-cell studies have come closer, including one that recently separated small-intestinal NETs (siNETs) into epithelial-like and neuronal-like subtypes (8). These studies, however, have been limited by modest cohorts, heterogeneous platforms, and dissociated tissue that lacks spatial context (8–10). Until recently, no study had profiled pancreatic (PanNETs) and siNETs at sufficient scale to compare the two sites directly or paired transcriptional states with chromatin accessibility and spatial position in the same specimens.

## Two malignant cell programs spanning a single continuum

In this issue of the *JCI*, Karam, Hoffman, and colleagues address that gap with the largest multimodal GEP-NET dataset assembled to date (11): they performed joint single-nucleus RNA-seq and assay for transposase-accessible chromatin sequencing (ATAC-seq) on 38 archival well-differentiated tumors (21 PanNETs and 17 siNETs), yielding 217,636 transcriptomes and 74,004 chromatin accessibility profiles, together with Visium spatial transcriptomics on 19 matched specimens and orthogonal Xenium imaging on 4 specimens. To avoid forcing cells into discrete types, the authors applied consensus nonnegative matrix factorization (cNMF) (12), an unsupervised approach that decomposes each tumor into gene expression programs a cell can express in continuous, overlapping amounts. Factorization was run per sample and aggregated across the cohort, which avoids mistaking patient-specific batch or copy number artifacts for biology, and resolved three malignant programs in siNETs and four in PanNETs, two of which were shared across both organ sites.

The first, a neuronal-like program (cNMF1), was defined by synaptic and axon guidance genes including *NRXN1*, *DLG2*, *RIMS2*, and *KCNJ3*. The second, a secretory neuroendocrine program (cNMF2), carried the canonical machinery of hormone production and release (*CHGA*, *CHGB*, *PCSK1*, and *SCG5*), with enrichment for oxidative phosphorylation and mTORC1 signaling. Two-dimensional density plots of program scores demonstrated that malignant cells populate a continuous distribution between the two states and do not partition into discrete subtypes. Individual tumors therefore differ in the proportion of malignant cells falling into each state.

## Organ-specific chromatin underlies shared programs

Despite this transcriptional convergence, the matched ATAC data showed that program-high cells in the two organ sites were enriched for largely different transcription factor motifs. In siNETs, neuronal-like cells showed accessibility at MAX:MYC, MITF, and CLOCK motifs and secretory cells at HOXA9, AP-1, and SOX9 motifs. In PanNETs, the same two programs were associated with different transcription factors: neuronal-like cells with TFAP4, NR3C2, and the islet fate regulator ISL1 (13) and secretory cells with a homeodomain set including MSX2, LBX2, and EN2. Motif accessibility tracked with expression of the corresponding predicted target genes in the matched PanNET transcriptomes, consistent with a regulatory relationship. A program score may therefore be portable across GEP-NETs even though the transcription factors sustaining it differ by site. If so, the pathways each malignant cell state engages are more likely to be shared therapeutic targets than the site-specific factors upstream.

## Malignant cell states occupy distinct tissue niches

Projected onto the Visium data, which read the whole transcriptome in 55 μm spots containing several cells each, revealed that the two programs were not randomly distributed in tissue. Secretory-high malignant spots sat somewhat deeper within the tumor interior, and neuronal-like–high spots were distributed somewhat closer to the periphery. Secretory-high regions localized to fibrovascular, stromally infiltrated areas with elevated *COL1A1*, *SPARC*, and *MMP2* showed significantly greater spatial association with endothelial and stromal compartments than neuronal-like–high regions. Furthermore, these secretory-high regions expressed *TGFB1* and *ITGB1* in a polarized fashion, with TGFB1–ITGB1, VEGFA–FLT1, and LAMA2–ITGA1 among the interactions predicted from the single-nucleus data. In contrast, neuronal-like–high regions occupied densely cellular, stromally sparse territory with predicted neuroimmune signaling to myeloid cells through CX3CL1/CX3CR1 and IL34 or CSF1 signaling via CSF1R. These regions also carried a significantly higher proportion of immune spots in their immediate neighborhood in both siNETs and PanNETs compared with secretory-high regions. The matched H&E sections showed the same distinction between programs, with little intervening stroma where the neuronal-like program dominated and stromal infiltration where the secretory program did. Xenium imaging, which resolves single cells but reads only a targeted gene panel, was applied to four specimens and placed endothelial, fibroblast, and myeloid cells immediately adjacent to malignant cells in secretory-high regions. Consistency across two platforms of differing resolution increased confidence that these associations reflect regional tissue organization.

Fibrosis is a defining clinical feature of NETs, ranging from mesenteric desmoplasia causing bowel obstruction to carcinoid heart disease. NET fibrosis has long been attributed to secreted vasoactive peptides and serotonin acting through TGF-β1 (14). The secretory program is a candidate cellular source of these signals because its siNET form includes *TAC1*, encoding substance P, and secretory-high regions expressed *TGFB1* within the fibrovascular niche. Karam et al.’s study did not examine whether tumors producing these mediators are the ones that develop desmoplastic complications, a question that existing cohorts could answer.

## Program composition tracks with clinical stage

By contrast, Karam et al. did directly examine disease stage. In both tumor types, neuronal-like scores were higher in localized primary tumors, and secretory scores were higher in metastatic lesions; within primary PanNETs, the neuronal-like program significantly exceeded the secretory one, while the primary versus metastatic differences did not survive correction for multiple comparisons in a discovery cohort containing only three PanNET metastases. An external cohort examined primary tumors only. Among 22 PanNET primaries from patients with localized or metastatic disease (15), those from patients with metastases carried significantly lower neuronal-like scores, with secretory scores directionally higher but not significant. The direction of relationships between dominant programs in primary versus metastatic tumors was consistent across datasets, but the individual comparisons are small, and these associations should be considered hypothesis generating.

## What this framework needs next

The most immediate question is whether the cell state axis predicts treatment benefit. GEP-NETs respond to antiangiogenic therapy more consistently than most solid tumors (16–18), and secretory program abundance can be scored from archival bulk RNA-seq, so existing trial biospecimens could test for greater benefit from sunitinib, surufatinib, or cabozantinib among secretory-high tumors. The same specimens could relate the program to somatostatin receptor 2 (SSTR2) expression and therefore to response to the SSTR2-targeted radionuclide therapy ^177^Lu-DOTATATE (19) and indicate whether myeloid-directed agents are better matched to neuronal-like–high tumors.

Beyond that, establishing whether the fibrovascular niche instructs the secretory state or whether the secretory state builds its own niche requires perturbation in models that the field does not yet reliably have (20). Longitudinal and on-treatment sampling are needed to determine whether malignant cells shift between neuronal and secretory states under therapeutic pressure or retain the composition seen at resection, ideally using technologies that deliver whole-transcriptome measurement at single-cell spatial resolution (21). Because this cell state axis is shared by PanNETs and siNETs and can be scored in archival tissue, its prognostic and predictive value can be tested in retrospective cohorts and completed trials.

## Manuscript information

### AI disclosure.

Claude (Anthropic), Fable 5.5, was used to edit the manuscript for accuracy and clarity; the author reviewed and takes full responsibility for the content.

### Conflict of interest.

The author has declared that no conflict of interest exists.

## Version 1 11/02/2026

Electronic publication
